# Background and descriptive features of rabies-suspected animals in Central Luzon, Philippines

**DOI:** 10.1186/s41182-021-00351-x

**Published:** 2021-07-28

**Authors:** Milagros R. Mananggit, Kazunori Kimitsuki, Nobuo Saito, Alyssa Marie G. Garcia, Patricia Mae T. Lacanilao, Joely T. Ongtangco, Cornelio R. Velasco, Maria Victoria D. Rosario, Maria Glofezita O. Lagayan, Kentaro Yamada, Chun-Ho Park, Satoshi Inoue, Motoi Suzuki, Mariko Saito-Obata, Yasuhiko Kamiya, Daria L. Manalo, Catalino S. Demetria, Beatriz P. Quiambao, Akira Nishizono

**Affiliations:** 1 Regional Animal Disease Diagnostic Laboratory, Department of Agriculture Field Office III, San Fernando, Pampanga Philippines; 2 Department of Microbiology, Faculty of Medicine, Oita University, Yufu, Oita Japan; 3 School of Tropical Medicine and Global Health, Nagasaki University, Nagasaki, Nagasaki Japan; 4 Bureau of Animal Industry, Quezon City, Philippines; 5 Laboratory of Veterinary Public Health, Department of Veterinary Medical Science, Faculty of Agriculture, University of Miyazaki, Miyazaki, Miyazaki Japan; 6 Department of Veterinary Pathology, School of Veterinary Medicine, Kitasato University, Towada, Aomori, Japan; 7 National Institute of Infectious Disease, Tokyo, Japan; 8 Center for Animal Disease Control, University of Miyazaki, Miyazaki, Miyazaki Japan; 9 Tohoku University Graduate School of Medicine, Sendai, Miyagi Japan; 10 Research Institute for Tropical Medicine, Muntinlupa City, Metro Manila Philippines

**Keywords:** Neglected tropical diseases, Rabies, Animal rabies case, Surveillance, Philippines

## Abstract

**Background:**

The Philippines is one of the major endemic countries for canine rabies in Southeast Asia. However, detailed description and analysis of laboratory-confirmed animal rabies are limited. Highly accurate surveillance requires a thorough understanding of the target area-specific problems and obstacles. Therefore, we aim to describe and analyze the rabies suspect animals in Central Luzon, Philippines, to clarify the characteristics of management and clinical signs by conducting interviews with the owners.

**Methods:**

We prospectively collected information on the rabies suspect animals submitted to the Regional animal laboratory in Central Luzon through passive laboratory-based rabies surveillance between 1st April 2019 and 30th September 2020. We performed active interviews directly or telephonically with the owner. The direct fluorescent antibody test was performed on the hippocampus, brain stem, and cerebellum for laboratory confirmation. Descriptive statistics were used to characterize the number of rabies cases according to management methods and characteristics of suspected animals during the observation period. Clinical symptoms of suspected rabid animals were analyzed by univariate logistic regression analysis.

**Results:**

There were 292 sample submissions during the study period. Of these, 160 were positive for dFAT. Samples of pet animals (85.3%) provided by owners or their acquaintances (59.2%) accounted for the majority of laboratory confirmed cases. Case mapping showed that more rabies-suspected cases were sent from areas near the regional laboratory than from those far from the laboratory, despite the incidence of rabies being high in these areas. The management and clinical symptoms of 227 animal cases showed that most owners were managing their animals at home and were allowing them to roam outside (69.6%) and be unvaccinated (78.9%). Rabid animals were more likely to manifest aimless running, restlessness, and agitation.

**Conclusions:**

Our study provided some features of animals with laboratory-confirmed rabies in Central Luzon. However, most of the samples were submitted from areas near the rabies diagnosis laboratory, and the number of samples submitted from remote areas was low. To improve the surveillance capacity, it is necessary to increase sample submissions from remote areas.

**Supplementary Information:**

The online version contains supplementary material available at 10.1186/s41182-021-00351-x.

## Background

Controlling the occurrence of rabies in domestic dogs is crucial to eradicate human rabies. Domestic dogs serve as major reservoirs of rabies and cause over 90% of human deaths from rabies worldwide [1, 2]. In the previous decades, there has been a dramatic decrease in the number of dog-mediated human rabies cases in the western hemisphere because of effective mass dog vaccination in addition to the control of the dog population [3–5]. As opposed to measurements of the estimated 59,000 annual human deaths due to dog-mediated rabies worldwide, the majority occur in Africa (36.4%) and Asia (59.6%) [6]. One of the contributing factors of rabies endemic to Asia and Africa is the limited capacity of surveillance in animal rabies. As a result, there is a lack of important epidemiological data essential for designing effective control strategies.

Philippines is one of the major endemic countries in Southeast Asia and has approximately 200–300 human cases annually [7]. In 2007, the Republic Act No. 9482, the Anti-Rabies Act of 2007, was implemented to prevent and control human rabies [7]. The target of the National Rabies Committee is to eliminate rabies by the year 2030. However, despite efforts to expand animal bite treatment centers nationwide and to provide free rabies post-exposure prophylaxis, there has not been a marked decrease in the incidence of human rabies in recent years. In particular, the incidence of human rabies cases in Central Luzon (Region III) was the highest between 2008 and 2018 [7].

Highly accurate surveillance requires a thorough understanding of target area-specific problems and obstacles. There is limited epidemiological data on canine rabies cases available in the Philippines. According to the investigation by Doming et al., which analyzed laboratory records on animal rabies examinations from 2002–2013 in Central Luzon, 89% of the samples came from dogs, and almost 80% of rabies-positive puppies were 3 months old and below [8]. However, no detailed information is available about who submitted the animal samples, vaccination history, and what symptoms manifest during the observation period. These data would improve the surveillance system and contribute to support toward national rabies control programs. The Regional Animal Disease Diagnostic Laboratory (RADDL) plays a principal role in the laboratory confirmation of animal rabies in the Philippines. However, limited information is available through laboratory surveillance because interviews often do not involve persons related to rabies cases, such as owners. Therefore, we conducted a prospective study in a regional animal laboratory (RADDL III) located in Central Luzon where the incidences of human and animal rabies were the highest in the country. In this study, we aimed to prospectively describe the epidemiological data for animal rabies in the Central Luzon (Region III). Furthermore, we investigated characteristics such as the management and clinical signs of laboratory-confirmed rabies animals.

## Methods

### Study site

**Fig. 1:**
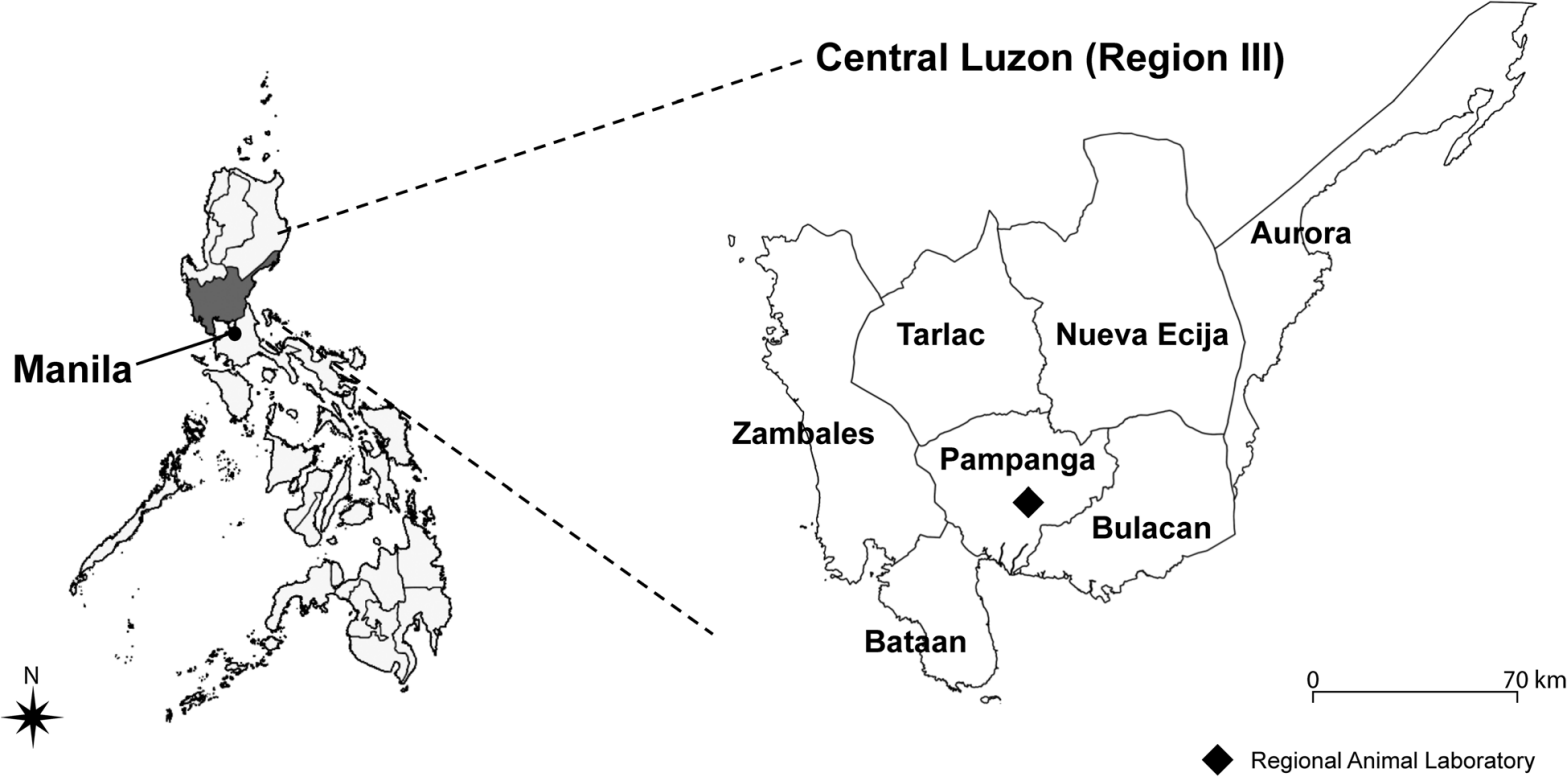
Map depicting the locations of Region III in the Philippines and Regional Animal Laboratories

We conducted a prospective observational study of suspected rabid animals to evaluate the diagnostic accuracy of the lateral flow device (LFD) and investigate the characteristics of rabies animals at RADDL III, a government agency in the Philippines. The details of the study design and setting have been previously described [9]. This regional laboratory receives suspected rabies specimens (heads or carcasses) from Central Luzon (Region III) (Fig. 1). Region III has a population of 11,218,177 [10]. In 2018, the number of human and animal rabies infections officially reported in Region III was 58 and 252, respectively, the highest in the Philippines [7]. In this study, we described data on suspected rabid animals that were submitted to this laboratory between 1st April 2019 and 30th September 2020.

### Data collection

We conducted semi-structured interviews to collect detailed information about the animal, owner, and bite victims using a standard questionnaire [8, 9]. The research staff interviewed, directly or telephonically, the owners or those who sent the animal head or carcass. We excluded cases if the information was not sufficient, such as cases where the owner refused to take part in the interview. Finally, we conducted a detailed analysis of the remaining cases.

### Laboratory confirmation

The reference diagnostic test, called the direct fluorescent antibody test (dFAT), was performed using the head or carcass of suspected rabid animals submitted by the sender. Briefly, touch impressions of small transverse sections (2–3 mm in thickness) of the hippocampus, brain stem, and cerebellum were stained with fluorescein isothiocyanate-conjugated anti-rabies monoclonal antibody (Fujirebio, Malvern, PA, USA; lot No. 309303) according to the standard operating procedure [8, 9]. The stained samples were examined under an epifluorescence microscope (E200, Nikon, Tokyo, Japan) to confirm the presence of the rabies virus antigen by two independent examiners.

### Data analysis

Study data were collected and managed using REDcap electronic data capture tools (REDcap Consortium, Nashville, TN, USA) hosted at the Nagasaki University and Oita University. All analyses were performed using STATA version 15.0 (StataCorp, TX, USA) or GraphPad Prism 8 (GraphPad Software, CA, USA). Descriptive statistics were used to characterize the number of rabies cases according to sex, age group, and spatial distribution of submitted cases. The age of the animal was compared according to the following groups: 0–3, 4–12, 13-24, 24–36, and > 36 months. Spatial patterns showing the number of submitted cases were mapped using the QGIS ver. 3.1.4 (QGIS development team, Open Source Geospatial Foundation Project). Clinical symptoms of suspected rabid animals were analyzed by univariate logistic regression analysis.

### Ethical statement

This study was approved by the Biosafety Clearance of Research Institute for Tropical Medicine (RITM) (No. 190116). We obtained verbal agreement and consent from the sender or animal owner. Finally, personal information of the owner or sender was encoded to avoid identification.

## Results

### Animal rabies surveillance in Central Luzon

**Fig. 2:**
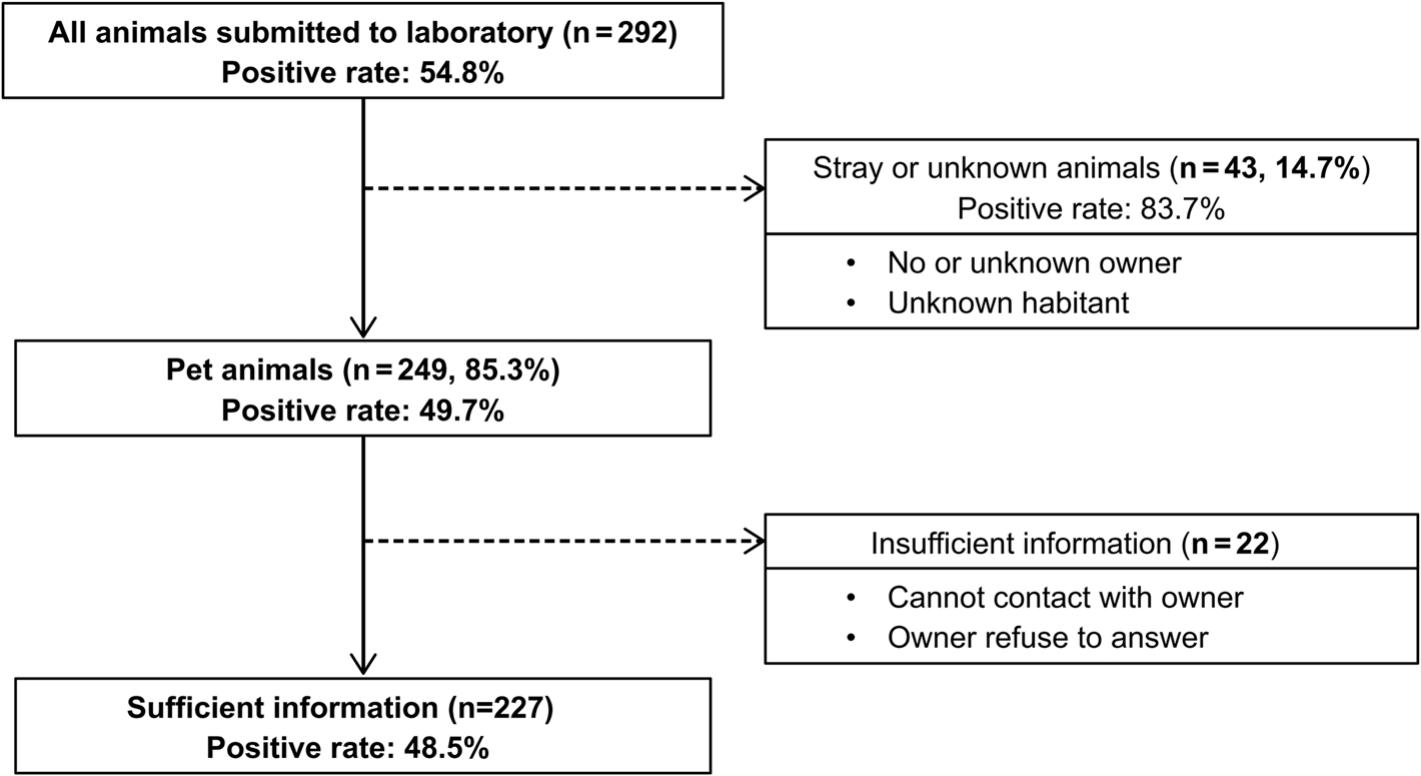
Number of animal samples submitted to Regional Animal Laboratory and the positive rate

**Fig. 3:**
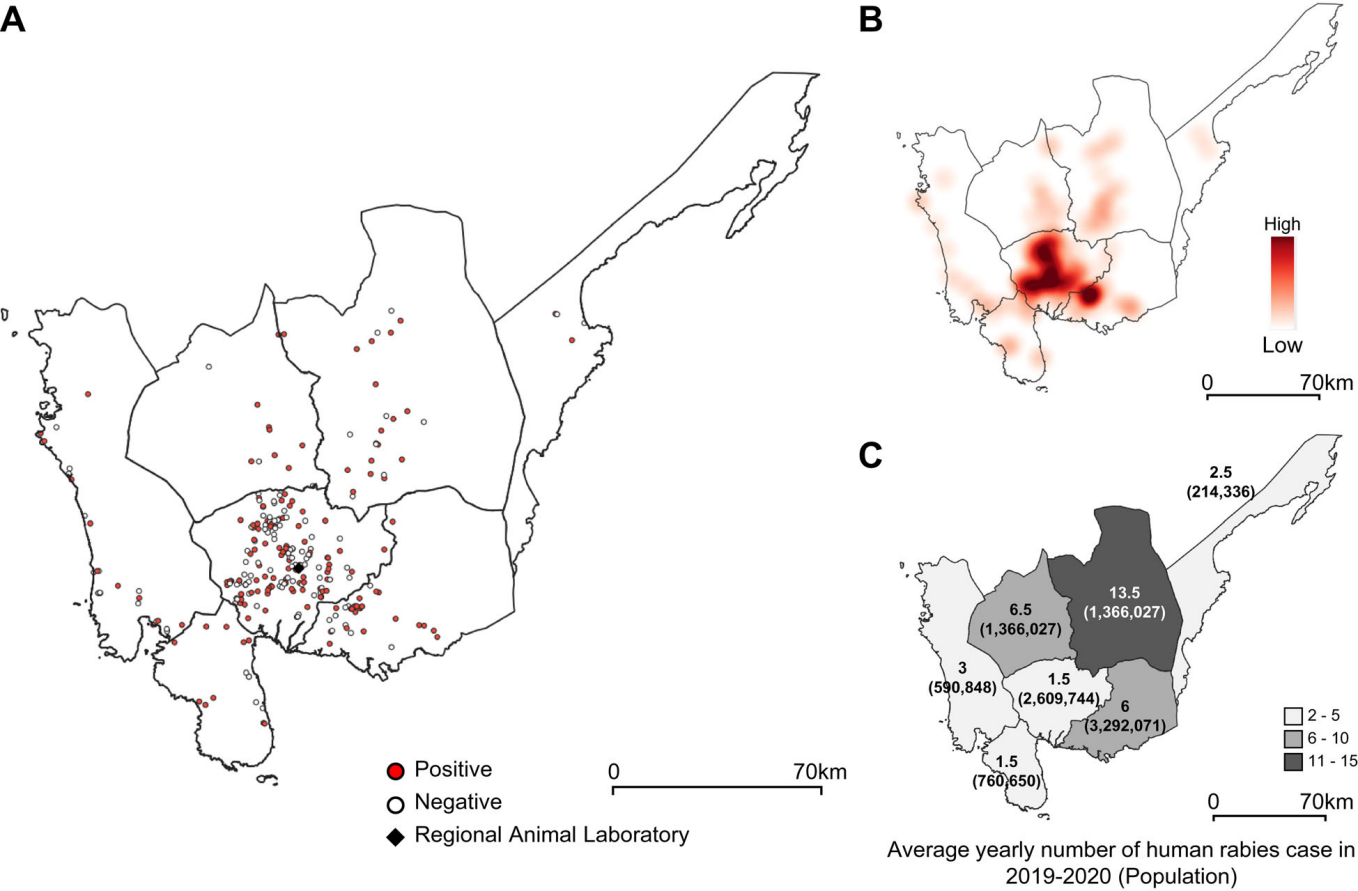
Distribution of animal and human rabies cases during the study period. (**A**) Distribution of the submission and result of the rabies cases in Region III. (**B**) Heat map indicates the density of positive cases in animal surveillance in Region III. (**C**) Color scale indicates the mean human rabies incidence and population per province

**Fig. 4:**
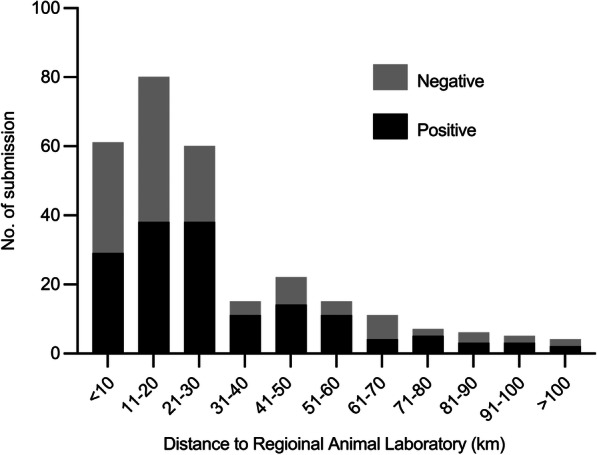
Distribution of sample submissions by distance

In this study, a total of 292 animal samples were tested, of which 160 and 132 samples tested positive and negative, respectively (Fig. 2). In terms of ownership, most samples were from pet animals (85.3%), while the rest were from stray or unknown animals (14.7%). The stray or unknown animals were more likely to test positive for rabies (83.7%) than the pet animals (49.7%) (*P* < 0.001) (Fig. 2). In general, the number of submissions was higher in areas within 30 km of RADDL III than areas far from RADDL III (Figs. 3A, B, and 4). The mean number of human rabies cases in Nueva Ecija was the highest from 2019 to 2020, while submitted cases of suspected rabid animals from Nueva Ecija were approximately 15.1% of that of Pampanga (Fig. 3C and Table 1). The species with the highest number of submitted cases and maximum positive rate was dogs (226 cases, 91.1%), while both the number of submissions and positive rate were relatively lower in cats than in dogs (25 cases, 8.6%) (Table 1). In terms of age, 38.0% of the samples were from animals aged < 3 months, and this age group was more likely to test negative for rabies than other age groups. The majority of animals belonged to households or neighborhoods, and 59.2% samples were submitted by owners (or their relatives) (Table 1). “This animal bit human” was the most common reason for submitting samples (91.5%) (Table 1).

**Table 1:** Characteristics of 292 submitted animal samples

	Total		Positive		Negative	
	Total	(%)	Total	(%)	Total	(%)
Species						
Dog	266	(91.1)	158	(69.9)	108	(47.8)
Cat	25	(8.6)	2	(8.0)	23	(92.0)
Other	1	(0.3)	0	(0.0)	1	(100.0)
Sex (*n* = 238)						
Male	136	(46.6)	64	(47.1)	72	(52.9)
Female	102	(34.9)	65	(63.7)	37	(36.3)
Age (*n* = 242)						
0-3 months	92	(38.0)	28	(30.4)	64	(69.6)
4-12 months	58	(24.0)	38	(65.5)	20	(34.5)
13-24 months	28	(11.6)	23	(82.1)	5	(17.9)
25-36 months	24	(9.9)	17	(70.8)	7	(29.2)
> 36 months	40	(13.2)	17	(42.5)	23	(57.5)
Body size						
Large (≧ 25 kg)	7	(2.4)	3	(42.9)	4	(57.1)
Middle (≧ 10 kg)	62	(21.2)	47	(75.8)	15	(24.2)
Small (< 10 kg)	223	(76.4)	110	(49.3)	113	(50.7)
Province						
Aurora	4	(1.4)	2	(50.0)	2	(50.0)
Bataan	17	(5.8)	10	(58.8)	7	(41.2)
Bulacan	38	(13.0)	27	(71.1)	11	(28.9)
Nueva Ecija	26	(8.9)	18	(69.2)	8	(30.8)
Pampanga	172	(58.9)	85	(49.4)	87	(50.6)
Tarlac	10	(3.4)	8	(80.0)	2	(20.0)
Zambales	25	(8.6)	10	(40.0)	15	(60.0)
Submitted by						
Owner (or related to owner)	173	(59.2)	77	(44.5)	96	(55.5)
Local Government *	76	(26.0)	53	(69.7)	23	(30.3)
Victim (related to victim) or neighbor	33	(11.3)	23	(69.7)	10	(30.3)
Animal hospital	3	(1.0)	2	(66.7)	1	(33.3)
Others	7	(2.4)	5	(71.4)	2	(28.6)
Reason(s) for the submission of the animal sample**						
This animal bit human	269	(91.5)	148	(55.0)	121	(45.0)
This animal showed the symptom of rabies	21	(7.1)	12	(57.1)	9	(42.9)
This animal was not suspected of rabies, but for investigation	1	(0.3)	0	(0.0)	1	(100.0)
Unknown	3	(1.0)	2	(66.7)	1	(33.3)

*Include National, Regional, Province, City, Municipal, or Barangay institute, **Multiple choice

### Features of management and clinical signs of laboratory-confirmed animal rabies

Out of 249 pet animals, the management strategy and clinical symptoms of 227 pets from owners are summarized in Tables 2 and 3, respectively. Most of the animals were managed freely or by households but allowed to roam outside (69.6%) and were bred with other animals (72.2%). According to the vaccination status, the majority of animals were unvaccinated (78.9%), and 15.0% had been vaccinated more than once; however, there were no significant differences in vaccination rate between positive and negative cases (Chi^2^ = 0.34; *p* = 0.56) (Table 2). Among vaccinated animals, 10, 7, and 1 were vaccinated once, multiple times, and unknown number of times, respectively. For the latest vaccinations, 2 were more than 3 years before, 11 cases were vaccinated within 2 years, and the timing was unknown in 5 cases (Additional file 1).

**Table 2:** Management method, characteristics of suspected animals during observation period, and number of victims of 227 suspected animals

	Total		Positive		Negative	
Descriptive feature	Total	(%)	Total	(%)	Total	(%)
***Management method***						
A movable range (*n* = 220)						
Confined	26	(11.5)	4	(15.4)	22	(84.6)
Leashed	36	(15.9)	19	(52.8)	17	(47.2)
Free	71	(31.3)	26	(36.6)	45	(63.4)
Household but roam outside	87	(38.3)	59	(67.8)	28	(32.2)
Management with other animals						
Yes	164	(72.2)	78	(47.6)	86	(52.4)
No	52	(22.9)	24	(46.2)	28	(53.8)
Unknown	11	(4.8)	8	(72.7)	3	(27.3)
Rabies vaccination (*n* = 213)						
More than once	34	(15.0)	18	(52.9)	16	(47.1)
Never	179	(78.9)	85	(47.5)	94	(52.5)
***Characteristics of suspected animals during observation period***						
Period of illness (day)						
0-5	145	(63.9)	78	(53.8)	67	(46.2)
6-10	34	(15.0)	16	(47.1)	18	(52.9)
11-15	8	(3.5)	1	(12.5)	7	(87.5)
> 15	12	(5.3)	1	(8.3)	11	(91.7)
Unknown	28	(12.3)	14	(50.0)	14	(50.0)
Contact with other animals last 14 days (*n* = 221)						
Yes	182	(80.2)	95	(52.2)	87	(47.8)
No	39	(17.2)	10	(25.6)	29	(74.4)
Showed suspected symptoms (*n* = 223)						
Yes	130	(57.3)	93	(71.5)	37	(28.5)
None	93	(41.0)	14	(15.1)	79	(84.9)
Cause of death						
Illness	176	(77.5)	75	(42.6)	101	(57.4)
Euthanasia	31	(13.7)	25	(80.6)	6	(19.4)
Accident	5	(2.2)	3	(60.0)	2	(40.0)
Dead at the time finding	12	(5.3)	5	(41.7)	7	(58.3)
Others	3	(1.3)	2	(66.7)	1	(33.3)
***Victim***						
No. of victim						
0	19	(6.5)	10	(9.1)	9	(7.7)
1	163	(55.8)	67	(60.9)	96	(82.1)
2	31	(10.6)	22	(20.0)	9	(7.7)
3	9	(3.1)	7	(6.4)	2	(1.7)
4	3	(1.0)	2	(1.8)	1	(0.9)
> 5	2	(0.7)	2	(1.8)	0	(0.0)

**Table 3:** Logistic regression model for clinical characteristics associated rabies suspected animals

	No. of positive (%) (***n*** = 93)		No. of negative (%) (***n*** = 37)		OR	95% CI	***P*** value
Restlessness	69	(74.2)	7	(18.9)	26.44	11.23-62.25	< 0.001
Agitation	37	(39.8)	2	(5.4)	29.14	6.82-124.6	< 0.001
Unprovoked aggressiveness	66	(71.0)	11	(29.7)	14.45	6.97-29.95	< 0.001
Aimless running	42	(45.1)	2	(5.4)	35.51	8.33-151.4	< 0.001
Eating inanimate objects	52	(55.9)	6	(16.2)	16.59	6.72-40.91	< 0.001
Drooling saliva	24	(25.8)	20	(54.0)	1.35	0.70-2.62	0.3682
Paralysis (hindleg/foreleg)	26	(27.9)	8	(21.6)	4.21	1.82-9.79	< 0.001
Paralysis (jaw/tongue)	11	(11.8)	2	(5.4)	6.39	1.38-29.52	0.051

The period of illness was 0–5 days, 6–10 days, 11–15 days, and over 15 days for 145 cases, 34 cases, 8 cases, and 12 cases, respectively. The duration of illness for the positive group was shorter than that of the negative group (median (range) = 2.0 days (1–4) vs 4 days (2–7), *p* < 0.001). The animals that tested positive had a history of contact with other animals 14 days before euthanasia or death (80.2%). During this observation period, rabies-positive animals showed suspected symptoms (Table 2). Rabid animals were more likely to manifest aimless running (OR = 35.51; 95% CI = 8.33-151.4), restlessness (OR = 26.44; 95% CI = 11.23-62.25), and agitation (OR = 29.14; 95% CI = 6.82-124.6) (Table 3).

## Discussion

In this study, we provide detailed information about canine rabies in a highly endemic area of the Philippines. Passive laboratory-based surveillance often fails to elucidate precise, essential data. However, our active interview survey revealed the epidemiological background of laboratory-confirmed cases. An important finding of our study is that there is remarkably limited surveillance data available for canine rabies in areas that are far from the regional laboratory. The majority of the animals that tested positive were owned but allowed to roam freely outside the house. The samples were submitted by the owner or an acquaintance of the owner. Additionally, the laboratory-confirmed rabid animals were mostly unvaccinated, bit humans during their illness, and were < 3 months of age. In addition, the rabies positive dogs where characterized by clinical symptoms such as restlessness, and these symptoms were reported by the owner as well.

We observed the highest number of positive cases and sample submission in Pampanga province where the regional animal laboratory is located (sample submissions per 100,000 people per year: Pampanga: 7.8, Aurora: 1.9, Bataan: 2.2, Bulacan: 1.1, Nueva Ecija: 1.2, Tarlac: 0.7, and Zambales: 4.2). However, more human rabies cases have been reported in the provinces far from the regional laboratory, such as Nueva Ecija, Tarlac, and Bulacan, than in Pampanga. Since human rabies is transmitted from dogs, there is a possibility that the number of rabies-positive dogs is high in areas where human rabies is endemic [1]. Therefore, we can assume that underreporting of animal rabies occurs in areas far from the laboratory. According to our survey, the majority of samples were submitted by the owner of the animal. We did not investigate the transportation means to the laboratory, but the sender usually uses a private vehicle or sometimes public transport (bus, jeep, and motorized tricycle). Use of public transport can be a burden, especially in areas far from the laboratory, and this might contribute to the underreporting and limited surveillance of animal rabies in remote areas. Underreporting and inadequate surveillance of animal rabies might exist in many rabies endemic areas worldwide. This leads to difficulties in expanding diagnostic facilities to these areas. One possible solution for the limited animal surveillance is to establish additional facilities to diagnose animal rabies. However, the current standard test, dFAT, is complicated and requires a laboratory equipped with a fluorescence microscope and trained staff. This is the main obstacle to the establishment of additional facilities. Simple, rapid, and low-cost methods such as LFD can be used to establish a diagnosis in a small laboratory in a remote area or in the field [9, 11]. This novel diagnosis can be a solution to promote animal surveillance.

Our study identified several features of laboratory-confirmed animal rabies in the Central Luzon. First, the majority rabid animals (85.3%) were owned and free-roaming, although this may be due to the selection bias in passive surveillance. Second, most of the positive animals were aged under 1 year, particularly puppies aged < 3 months. Although vaccination for dogs under 3 months of age is recommended by the WHO and the national program in the Philippines [1, 7], routine vaccination of puppies under 3 months of age is not performed in the area. Therefore, strengthening mass vaccination for puppies should be considered in this area [8]. Third, we observed that 18 rabies positive animals were reported to be vaccinated, although the majority of the laboratory-confirmed animals were not vaccinated. Our study could not identify vaccine registration, and there might be recall bias. We could not obtain detailed information such as vaccine type, detailed vaccination time, and blood neutralizing antibody titer [12]. Further assessment of rabid animals with vaccine history is needed.

Concerning the reason for submission, “this animal bit human” was more common than “this animal showed symptoms of rabies.” This result suggests that bite incidence might be the most important trigger for rabies investigation, as shown in other studies [13]. Regarding the symptoms, we identified some clinical symptoms in rabies-positive animals that were observed and reported by owners. These symptoms were restlessness, agitation, unprovoked aggressiveness, eating inanimate objects, and paralysis (hindlimb/forelimb). Tepsumethanon et al. proposed 6 criteria to distinguish rabies animals from other diseases (especially canine distemper) such as age, state of health of the dog, illness of evolving, clinical course in the last 3–10 days, presence of circling, and clinical signs. Diagnosis based on these criteria had a sensitivity and specificity of 90.2% and 96.2%, respectively [14]. In our study, clinical signs were confirmed by owners, implying that rabid animals can be identified in the community when people are aware of the clinical symptoms of rabies.

Our study has several limitations. First, our survey used submitted samples through a routine passive surveillance system. Therefore, our results do not show the true incidence of animal rabies in the area. Second, our survey period was only 18 months, which led to a difficulty in determining the seasonal variation in incidence [8, 15–18]. Third, recall bias may exist regarding vaccine history and clinical symptoms because we obtained this information through direct or telephonic interviews with owners. We conducted this study at a single regional animal laboratory in a region where the rabies incidence is higher than in other areas. Therefore, our results may not fully represent the characteristics of animal rabies in the country. Further research is required to clarify whether the distance to the diagnostic laboratory affects sample submission. Furthermore, our survey did not include certain samples from suspected rabid animals from the region because some owners submitted samples to other regional or central animal laboratories. In particular, people living in areas close to the border of Metro Manila are likely to submit samples to the central laboratory located in Metro Manila.

## Conclusions

Our study revealed that majority of the rabid animals were owned but allowed to roam freely outside the house. They were more likely to manifest aimless running, restlessness, and agitation. However, most of the data were from the samples submitted from areas near the rabies diagnosis laboratory. Reports of rabid animals from the remote areas were low, despite the high incidence of human rabies, suggesting that animal rabies surveillance in these areas was lacking. To improve the surveillance capacity, the number of sample submissions from the remote areas should be increased.

## Supplementary Information

**Additional File 1.** Details of vaccination history in confirmed animals.

**Additional File 2.** Specimen Information Sheet.

## Data Availability

The datasets used and/or analyzed during the current study are available from the corresponding author on reasonable request.

## Abbreviations

RADDL: Regional Animal Disease Diagnostic Laboratory
LFD: Lateral flow device
RITM: Research Institute for Tropical Medicine
