# Supplementary material for: Background and descriptive features of rabies-suspected animals in Central Luzon, Philippines
Source: Trop Med Health. 2021 Jul 28;49:59. doi: 10.1186/s41182-021-00351-x (PMC8320061; doi:10.1186/s41182-021-00351-x)
Supplement: Supplementary file 2 — Additional File 2. Specimen Information Sheet. [file 41182_2021_351_MOESM2_ESM.pdf]

RADDL3 ID.	JAPOHR Study ID	Interview by:

Specimen Information

Ver. 1.0 last undated on April 15, 2019

Sender is the owner?	☐ Yes → **Proceed to 2. Owner Information**<br>☐ No → **Proceed to 1. Sender Information**

1. Sender Information

Name of Sender		Age		Sex	☐ M ☐ F
Telephone Number					
Send from	☐ Dog Impounding (______________)<br>☐ Neighborhood<br>☐ Barangay Official (_______________)<br>☐ City veterinary office (_______________)<br>☐ Others (______________________)	☐ Victim<br>☐ Animal Hospital (_______________)<br>☐ City agricultural office (_______________)<br>☐ Provincial veterinary office (_______________)			
Address	Region	☐ Region III<br>☐ Other (____)	Province		
	City/Municipal		Barangay		
Owner Information	☐ Either the owner information or contact number is known.<br>→**Proceed to 2. Owner Information**<br>☐ Neither the owner information or contact number is known.<br>☐ This is Stray dog.<br>→**Proceed to 3. Animal Information**				

2.Owner Information

Interview Permission	☐ Yes<br>☐ No<br>☐ Unable to contact	Interview Type	☐ Direct Interview to the owner<br>☐ Sender Interview<br>☐ Telephone Interview to the owner		
Name of Owner		Age		Sex	☐ M ☐ F
Telephone Number					
Address	Region	☐ Region III<br>☐ Other (____)	Province		
	City/Municipal		Barangay		

Pet owned (How many)		☐ None ☐ Dog (___) ☐ Cat (___) ☐ Other (specify)______________(___)<br>Ownership methods of their dogs (Household Pet (__) / Free roaming (__) / Household but can be free roaming(__)))
	Vaccine History	☐ No vaccination for all dog pets ☐ Unknown<br>☐ No less than one dog was vaccinated<br>☐ (Animal1) Rabies vaccination Yes / No / Unknown<br>☐ (Animal2) Rabies vaccination Yes / No / Unknown<br>☐ (Animal3) Rabies vaccination Yes / No / Unknown<br>☐ (Animal4) Rabies vaccination Yes / No / Unknown<br>☐ (Animal5) Rabies vaccination Yes / No / Unknown<br>☐ (Animal6) Rabies vaccination Yes / No / Unknown
Occupation	Number: ________	(Occupation: ______________________________________)
Level of education / Highest degree of education		☐ None ☐ Primary ☐ Secondary ☐ Tertiary<br>☐ Vocational ☐ Unknown ☐ Other (specify) _______________<br>☐ Refuse to answer
How many people living together		
Monthly Income of the household (PHP)		☐ ≦5,000 ☐ 5,000-≦10,000 ☐ 10.000-≦20,000<br>☐ 20,000-≦30,000 ☐ 30,000-≦40,000 ☐ 40,000-≦50,000<br>☐ 50,000-≦60,000 ☐ ≧60,000 ☐ Unknown<br>☐ Refuse to answer

RADDL3 ID.	JAPOHR Study ID	Interview by:

3.Animal Information

Reason (s) brought this animal	☐ Bites human ☐ Showed the symptom of rabies<br>☐ Not showed the symptom of rabies, but suspect<br>☐ Not suspected rabies, but for investigation<br>☐ Found dead ☐ Others (____________)
Location of Find	☐ Household ☐ Neighborhood ☐ Public Area ☐ Other (specify)________<br>Region: ☐ Region III ☐ Other (____) — Province:<br>City/Municipal: — Barangay:<br>GPS coordinate: /
Specimen	☐ Whole carcass<br>☐ Head<br>☐ Brain<br>☐ Others (specify) ________<br>Received by: ____________
Transport of Storage of Specimen (Multiple choice)	☐ Unpreserved (>3 hours at room temperature)<br>☐ Fresh ☐ With ice<br>☐ Frozen ☐ Formalin fixed<br>☐ 50% buffered glycerol
Residence of the animal for the last 15 days	☐ Home ☐ Stray ☐ Unknown
Species	☐ Dog<br>☐ Cat<br>☐ Other ( )
Breed	☐ Mutt ☐ Breed ☐ Unknown<br>If Breed,<br>☐ Shih Tzu ☐ Chihuahuas ☐ Pomeranian<br>☐ Siberian Husky ☐ Beagles ☐ Labrador Retriever<br>☐ Chow Chow ☐ Pugs ☐ Poodle<br>☐ German Shepherd ☐ Other ( )
Age	☐ <1 month ☐ ≧1 month ☐ ≧2 months ☐ ≧3 months<br>☐ ≧4 months ☐ ≧5months ☐ ≧6 months ☐ ≧1year<br>☐ ≧2years ☐ ≧3 years ☐ Unknown<br>(__________month old (s))
Sex	☐ M ☐ F<br>☐ Unknown
Type of Ownership	☐ Household pet ☐ Pet of Neighbor<br>☐ Stray animal ☐ Unknown
Pet Management	☐ Confined ☐ leashed<br>☐ Free ☐ Unknown<br>☐ Household but roam outside
Body size	☐ Large (≧25kg) ☐ Middle (≧10kg) ☐ Small (<10kg)
Color	☐ White ☐ Black<br>☐ Brown ☐ Other ( )
Other Special Appearance / Characteristics	
Cause of Death	☐ Euthanasia (by__________) ☐ Illness<br>☐ Accident ☐ Others (______) ☐ Dead at the time finding<br>Date (dd/mm/yyyy)________ (Time)________
Rabies Vaccination History	☐ Yes ☐ No ☐ Unknown<br>If Yes, vaccination is<br>☐ 1st time ☐ 2nd time ☐ 3rd time<br>☐ 4th time ☐ More ( ) ☐ Unknown but every year<br>Latest Rabies vaccine history:<br>Type________, Date of last vaccination (dd/mm/yyyy)________<br>Place ____________
Contact with other animals?	☐ Yes ☐ No ☐ Unknown<br>If Yes, where :☐Household ☐Neighborhood ☐Other ☐Unknown
Health Condition of Biting Animal	☐ Healthy ☐ Sick ☐ Unknown<br>If sick, from (dd/mm/yyyy)______to (dd/mm/yyyy)________
Behavioral Changes (Multiple choice)	☐ None<br>☐ Restlessness<br>☐ Apprehensive watchful look<br>☐ Unprovoked Aggressiveness<br>☐ Aimless Running<br>☐ Eating inanimate objects<br>☐ Drooling saliva<br>☐ Paralysis (Hindleg/Foreleg)<br>☐ Paralysis (Jaw/Tongue)<br>☐ Unknown
Other Signs of Illness (Multiple choice)	☐ None<br>☐ Diarrhea<br>☐ Vomiting<br>☐ Inappetence<br>☐ Jaundice<br>☐ Skin lesions<br>☐ Lethargy/weakness<br>☐ Nasal/ Ocular discharge<br>☐ Convulsion /Seizures<br>☐ Others (specify)________<br>☐ Unknown
Bitten site (Multiple choice)	☐ None ☐ Head ☐ Neck ☐ Trunk<br>☐ Forelimb ☐ Hindlimb ☐ Other ( ) ☐ Unknown
Contact with human (last 15 days) (Multiple choice)	☐ Owner (s) ☐ Family ☐ Neighbor (s) ☐ Veterinarian<br>☐ Other ( )
How many victims?	☐ None → **Proceed to 5. Result of Examination**<br>☐ 1 ☐ 2 ☐ 3 ☐ More (__) →**Proceed to 4. Victim Information**

RADDL3 ID.	JAPOHR Study ID	Interview by:

Decapitated by	☐ Owner<br>☐ Victim<br>☐ Animal hospital veterinarian<br>☐ City veterinarian<br>☐ Provincial veterinarian	☐ Acquaintance of the owner<br>☐ Staff of dog Impounding<br>☐ Barangay Official<br>☐ City veterinarian<br>☐ Others (______________________)

RADDL3 ID.	JAPOHR Study ID	Interview by:

4. Victim Information

Victims Information 1

Interview Permission	☐ Yes<br>☐ No<br>☐ Unable to contact	Interview Type	☐ Direct Interview to the victim<br>☐ Sender Interview<br>☐ Telephone Interview to the victim		
Age		Sex	☐ M ☐ F		
Telephone Number					
Address	Region	☐ Region III<br>☐ Other (____)	Province		
	City/Municipal		Barangay		
Pre-exposure Rabies Vaccination Given	☐ Yes ☐ No ☐ Unknown				
Date of Bite (dd/mm/yyyy)		Time of Bite			
Site of Bite (Multiple choice)	☐ Yes ☐ None<br>☐ Unknown<br>If yes,<br>☐ Head ☐ Upper extremity<br>☐ Trunk ☐ Back<br>☐ Lower extremity<br>☐ Other (specify)____________<br>☐ Unknown	Nature of Bite (Multiple choice)	☐ Scratch<br>☐ Multiple ☐ Single<br>☐ Bad ☐ Moderate ☐ Severe<br>☐ Lick ☐ Unknown		
Bite Provoked?	☐ Yes ☐ No ☐ Unknown<br>☐ Yes ☐ No ☐ Unknown				
Location of Bitten	☐ Household ☐ Neighborhood ☐ Public area ☐ Other (specify)__________<br>☐ Unknown				
	Region	☐ Region III<br>☐ Other (____)	Province		
	City/Municipal		Barangay		
	GPS coordinate	/			
Initial Treatment	☐ Traditional healer / tandoc (Name of facility______________________)<br>specific the treatment (_____________________)<br>☐ Wound washing ☐ Stone ☐ Garlic ☐ Papaya soap<br>☐ Horn ☐ Sucking ☐ Herbal ☐ None<br>☐ Other (specify_____________________)				
Did you go to the medical practitioner (ABTC)?	☐ Yes<br>☐ No<br>If yes, facility is<br>______________	Treatment Received in the Facility (Multiple choice)	☐ HRIG ☐ ERIG<br>☐ 1st Anti-rabies Vac ☐ 2nd Anti-rabies Vac<br>☐ 3rd Anti-rabies Vac ☐ Tetanus<br>☐ Unknown ☐ None<br>☐ Others (specify)____________________		

Victims Information 2

Interview Permission	☐ Yes<br>☐ No<br>☐ Unable to contact	Interview Type	☐ Direct Interview to the victim<br>☐ Sender Interview<br>☐ Telephone Interview to the victim		
Age		Sex	☐ M ☐ F		
Telephone Number					
Address	Region	☐ Region III<br>☐ Other (____)	Province		
	City/Municipal		Barangay		
Pre-exposure Rabies Vaccination Given	☐ Yes ☐ No ☐ Unknown				
Date of Bite (dd/mm/yyyy)		Time of Bite			
Site of Bite (Multiple choice)	☐ Yes ☐ None<br>☐ Unknown<br>If yes,<br>☐ Head ☐ Upper extremity<br>☐ Trunk ☐ Back<br>☐ Lower extremity<br>☐ Other (specify)____________<br>☐ Unknown	Nature of Bite (Multiple choice)	☐ Scratch<br>☐ Multiple ☐ Single<br>☐ Bad ☐ Moderate ☐ Severe<br>☐ Lick ☐ Unknown		
Bite Provoked?	☐ Yes ☐ No				
Location of Bitten	☐ Household ☐ Neighborhood ☐ Public area ☐ Other (specify)__________				
	Region	☐ Region III	Province		

RADDL3 ID.	JAPOHR Study ID	Interview by:

		☐ Other (____)		
	City/Municipal		Barangay	
	GPS coordinate	/		
Initial Treatment	☐ Traditional healer / tandoc (Name of facility________________________) specific the treatment (______________________)<br>☐ Wound washing ☐ Stone ☐ Garlic ☐ Papaya soap<br>☐ Horn ☐ Sucking ☐ Herbal ☐ None<br>☐ Other (specify____________________)			
Did you go to the medical practitioner (ABTC)?	☐ Yes<br>☐ No<br>If yes, facility is<br>______________	Treatment Received in the Facility (Multiple choice)	☐ HRIG ☐ ERIG<br>☐ 1st Anti-rabies Vac ☐ 2nd Anti-rabies Vac<br>☐ 3rd Anti-rabies Vac ☐ Tetanus<br>☐ Unknown ☐ None<br>☐ Others (specify)_____________________	

Victims Information 3

Interview Permission	☐ Yes<br>☐ No<br>☐ Unable to contact	Interview Type	☐ Direct Interview to the victim<br>☐ Sender Interview<br>☐ Telephone Interview to the victim	
Age			Sex	☐ M ☐ F
Telephone Number				
Address	Region	☐ Region III<br>☐ Other (____)	Province	
	City/Municipal		Barangay	

Pre-exposure Rabies Vaccination Given	☐ Yes ☐ No ☐ Unknown		
Date of Bite (dd/mm/yyyy)		Time of Bite	
Site of Bite (Multiple choice)	☐ Yes ☐ None<br>☐ Unknown<br>If yes,<br>☐ Head ☐ Upper extremity<br>☐ Trunk ☐ Back<br>☐ Lower extremity<br>☐ Other (specify)____________<br>☐ Unknown	Nature of Bite (Multiple choice)	☐ Scratch<br>☐ Multiple ☐ Single<br>☐ Bad ☐ Moderate ☐ Severe<br>☐ Lick ☐ Unknown
Bite Provoked?	☐ Yes ☐ No		

Location of Bitten	☐ Household ☐ Neighborhood ☐ Public area ☐ Other (specify)__________			
	Region	☐ Region III<br>☐ Other (____)	Province	
	City/Municipal		Barangay	
	GPS coordinate	/		
Initial Treatment	☐ Traditional healer / tandoc (Name of facility________________________) specific the treatment (______________________)<br>☐ Wound washing ☐ Stone ☐ Garlic ☐ Papaya soap<br>☐ Horn ☐ Sucking ☐ Herbal ☐ None<br>☐ Other (specify____________________)			
Did you go to the medical practitioner (ABTC)?	☐ Yes<br>☐ No<br>If yes, facility is<br>______________	Treatment Received in the Facility (Multiple choice)	☐ HRIG ☐ ERIG<br>☐ 1st Anti-rabies Vac ☐ 2nd Anti-rabies Vac<br>☐ 3rd Anti-rabies Vac ☐ Tetanus<br>☐ Unknown ☐ None<br>☐ Others (specify)_____________________	

RADDL3 ID.	JAPOHR Study ID	Interview by:

5. Result of Examination

5-1. Sample collection

Date of Sampling (dd/mm/yyyy)				Save Picture	☐ Yes ☐ No
Facility	☐ RADDL3<br>☐ RITM	Examined by		Position	

Sample condition	Brain	☐ Acceptable (Fit for examination: brain is still intact and parts are still identifiable)<br>☐ Unacceptable (unfit for examination: decomposed, containing bacteria, liquefied, smashed, rotten, bloody, autolytic, dried out, desiccated, tacky, decaying, loss of structure)<br>☐ Not collected (Reason: ______)
	FSC	☐ Acceptable (Fit for examination: Still intact and parts are identifiable)<br>☐ Unacceptable (unfit for examination: decomposed, containing bacteria, liquefied, smashed, rotten, bloody, autolytic, dried out, desiccated, tacky, decaying, loss of structure)<br>☐ Not collected (Reason: ______)

5-2. Results (Brain)

☐	DME	Date of the Examination (dd/mm/yyyy)				Time	
		Facility	☐ RADDL3<br>☐ RITM	Examined by		Position	
		Result	Negative / Positive / Not perform / Inconclusive result			Save Picture	☐ Yes ☐ No
☐	dFAT	Date of the Examination (dd/mm/yyyy)				Time	
		Facility	☐ RADDL3<br>☐ RITM	Examined by		Position	
		Result	Negative / Positive / Not perform / Inconclusive result			Save Picture	☐ Yes ☐ No
☐	ICT (A)	Date of the Examination (dd/mm/yyyy)				Time	
		Facility	☐ RADDL3<br>☐ RITM	Examined by		Position	
		Result	Negative / Positive / Inconclusive result			Save Picture	☐ Yes ☐ No
☐	ICT (B)	Date of the Examination (dd/mm/yyyy)				Time	
		Facility	☐ RADDL3<br>☐ RITM	Examined by		Position	
		Result	Negative / Positive / Inconclusive result			Save Picture	☐ Yes ☐ No
☐	ICT (C)	Date of the Examination (dd/mm/yyyy)				Time	
		Facility	☐ RADDL3<br>☐ RITM	Examined by		Position	
		Result	Negative / Positive / Inconclusive result			Save Picture	☐ Yes ☐ No
☐	IHC	Date of the Examination (dd/mm/yyyy)				Time	
		Facility	☐ RADDL3<br>☐ RITM	Examined by		Position	
		Result	Negative / Positive / Inconclusive result			Save Picture	☐ Yes ☐ No
☐	qPCR	Date of the Examination (dd/mm/yyyy)				Time	
		Facility	☐ RADDL3<br>☐ RITM	Examined by		Position	
		Result	Negative / Positive (Ct value (___)) / Inconclusive result			Save data	☐ Yes ☐ No

5-3. Results (FSC)

☐	ICT	Date of the Examination (dd/mm/yyyy)				Time	
		Facility	☐ RADDL3<br>☐ RITM	Examined by		Position	
		Result	Negative / Positive / Inconclusive result			Save Picture	☐ Yes ☐ No
☐	IHC	Date of the Examination (dd/mm/yyyy)				Time	
		Facility	☐ RADDL3<br>☐ RITM	Examined by		Position	
		Result	Negative / Positive / Inconclusive result			Save Picture	☐ Yes ☐ No
☐	qPCR	Date of the Examination (dd/mm/yyyy)				Time	
		Facility	☐ RADDL3<br>☐ RITM	Examined by		Position	
		Result	Negative / Positive (Ct value ( )) /Inconclusive result			Save data	☐ Yes ☐ No

RADDL3 ID.	JAPOHR Study ID	Interview by:
